# Analyses of domains and domain fusions in human proto-oncogenes

**DOI:** 10.1186/1471-2105-10-88

**Published:** 2009-03-17

**Authors:** Qi Liu, Jinling Huang, Huiqing Liu, Ping Wan, Xiuzi Ye, Ying Xu

**Affiliations:** 1 Computational Systems Biology Laboratory, Department of Biochemistry and Molecular Biology, Institute of Bioinformatics, University of Georgia, Athens, GA 30602, USA; 2 Zhejiang California International Nanosystems Institute, Zhejiang University, Hangzhou, 310029, PR China; 3 Department of Biology, East Carolina University, Greenville, NC 27858, USA; 4 College of Computer Science, Zhejiang University, Hangzhou, 310027, PR China; 5 College of Life Science, Capital Normal University, Beijing, 100037, PR China

## Abstract

**Background:**

Understanding the constituent domains of oncogenes, their origins and their fusions may shed new light about the initiation and the development of cancers.

**Results:**

We have developed a computational pipeline for identification of functional domains of human genes, prediction of the origins of these domains and their major fusion events during evolution through integration of existing and new tools of our own. An application of the pipeline to 124 well-characterized human oncogenes has led to the identification of a collection of domains and domain pairs that occur substantially more frequently in oncogenes than in human genes on average. Most of these enriched domains and domain pairs are related to tyrosine kinase activities. In addition, our analyses indicate that a substantial portion of the domain-fusion events of oncogenes took place in metazoans during evolution.

**Conclusion:**

We expect that the computational pipeline for domain identification, domain origin and domain fusion prediction will prove to be useful for studying other groups of genes.

## Background

An *oncogene *is a modified gene that promotes unregulated proliferation of cells, increasing the chance that a normal cell develops into a tumor cell, possibly resulting in cancer [1]. The normal copy of such a gene is called a *proto-oncogene*. The first oncogene, *SRC*, was discovered in a chicken retrovirus in 1970 [2]. Since then, numerous oncogenes have been identified and classified into different groups based on their cellular functions. As of now, oncogenes have been identified at all levels of signal transduction cascades that control cell growth, proliferation and differentiation [1-3].

Protein domains are compact and semi-independent units of a protein, each of which may consist of one or more contiguous segments of a peptide chain and have its own biological function [3]. They are generally viewed as the basic unit of protein function and evolution. Various sequence- and structure-based methods have been developed for the identification of protein domains [4-6], and several domain databases, such as DALI [7], PFAM [8], SMART [9] and Prodom [10], have been established.

Recent studies on oncogenes and cancer pathology have pointed to the importance of individual domains and domain fusions in oncogenesis. It has been reported that genes containing domains from specific domain families may have particular relevance to human cancer [11-13]. For example, the tyrosine kinase domain is known to play significant roles in the development of numerous diseases such as cancer [11]. Another example is the ATM-related domain that is required for histone acetyltransferase recruitment and Myc-dependent oncogenesis [12]. Additionally, CML, a form of leukaemia, is associated with the fusion of *Bcr *and *Abl *genes or their constituent domains [13]. Therefore, understanding the constituent domains of oncogenes as well as their origins may shed new light about the initiation and development of cancers.

In this study, we have developed an integrated computational pipeline for studying the domain composition, domain fusion and domain origin. Specifically, our computational pipeline includes the following key components: (1) identification of the origin of each component domain of known oncogenes and the relevant fusion events; (2) co-occurrence analysis of oncogene domains; (3) identification of the domains and domain pairs that appear more frequently in oncogenes than in the background, namely the collection of all human genes; and (4) functional analyses of the identified frequent domains and domain pairs. We then applied this pipeline to all well characterized human oncogenes, and had a number of new and interesting observations. To the best of our knowledge, this is the first comprehensive analysis specifically addressing the domain composition, origin and fusion of oncogenes.

## Results and discussion

Using the computational procedures outlined in *Material and Methods*, we have carried out a detailed analysis of oncogene domains and co-occurring domains for their origins and functional analysis.

### A. Origin of oncogene domains

#### Origin of distinct domains in cellular organisms

**Figure 1:**
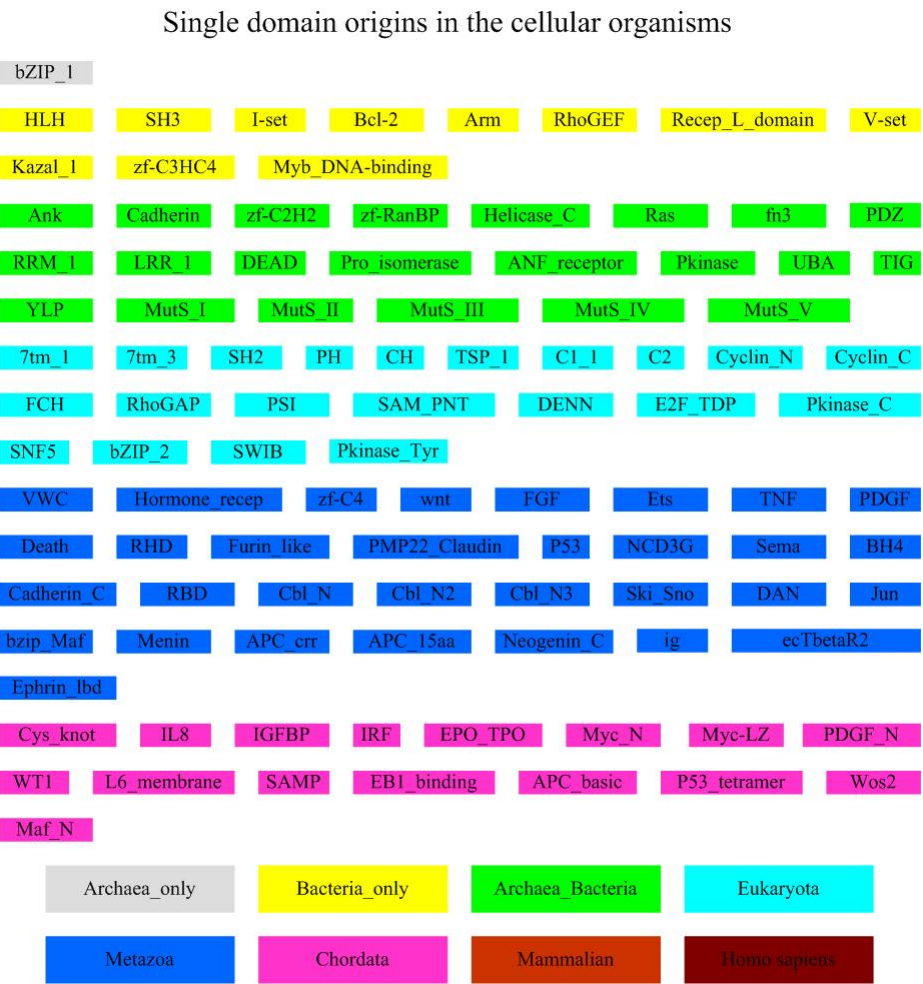
**Distributions of origins of 105 oncogene domains across cellular organisms**. Archaea: 1(1%); Bacteria: 17 (16%), Archaea_Bacteria: 22 (21%); Eukaryota: 19 (18%); Metazoa: 30(29%); Chordata: 16 (15%); Mammalia: 0 (0%); Homo sapiens: 0 (0%).

103 distinct domains [see Additional file 1] have been identified from 124 oncogenes, based on Pfam domain assignments. We have considered the subtype scenarios for specific domains, *i.e*., the different alignments for a specific domain in one clan and using one domain ID to denote the corresponding subtypes. In our dataset, there exist two alignments SH3_2 and SH3_1 for the SH3 domain. The same holds for the SAM domain, where SAM_PNT is the entry for the SAM domain and two different alignments, SAM_1 and SAM_2, exist for this entry, respectively. Although they have different accession numbers in Pfam, we just use SH3 and SAM_PNT to denote these two types of domains, respectively. The distribution of these domains' origins across different cellular organisms is given in Figure 1. About 50% (55/103) of oncogene domains have their origins in the early stages of organismal evolution prior to the emergence of the metazoans, and no domains are found to arise from mammals. It should be noted that these results have been further refined by our literature survey from the original *subtractive searching *results (see *Material and Methods*), to take potential HGT into consideration. Based on the literature search, we found that domain SWIB and non-enzymatic domains ig and SAM are likely to have arisen in eukaryote. Their homologs are identified in prokaryotes, likely resulted from HGTs from eukaryotes [14]. Also the origin of tyrosine kinases (Pkinase_Tyr) is probably in eukaryote and their presence in bacteria may also be explained most parsimoniously by HGT events [14].

In order to further analyze the statistical difference between the domain origin distribution of oncogenes *versus *that of the other genes, we have compared our results with Lipika *et al*. [15], which presented an analysis on the origins of the conserved domains in the whole human proteome. Table 1 presents a thorough calculation of the enrichment ratios of oncogene domains that originated from 8 categories (*i.e*., Bacteria_only, Archaea_only, Bacteria_archaea, low level Eukaryotes, Metazoan, Chordate, Mammalian, *Homo sapiens*) compared with the whole domain dataset in the human genes and their *p*-values. Our results indicate that the origin distribution of oncogene domains is largely consistent with that reported by Lipika *et al*. [15] for the whole human proteome, EXCEPT FOR those of bacterial or metozoan origins.

**Table 1:** Enrichment analysis of oncogene domain origination distribution compared with background human genome.

	*m*_s_	*M*	*n*_s_	*N*	*e-ratio*	*p-value*
**Bacteria_only**	**11**	**103**	**15394**	**88025**	**0.6107**	**3.96E-02**

Archaea_only	1	103	1021	88025	0.8370	6.64E-01

Bacteria_archaea	20	103	13052	88025	1.3095	1.13E-01

Lower-level Eukaryote	21	103	23391	88025	0.7673	9.26E-02

**Metazoan**	**32**	**103**	**15324**	**88025**	**1.7846**	**4.87E-04**

Chordate	16	103	13309	88025	1.0274	4.26E-01

Mammalian	0	103	4553	88025	0	0

Homo sapiens	0	103	1381	88025	0	0

Prokaryote	34	103	29367	88025	0.9894	5.16E-01

Eukaryote	69	103	58658	88025	1.0053	4.46E-01

#### Domain functions

We divided the oncogene domains into groups based on their GO annotation (Table 2). These oncogene domains show diverse functions, including regulation of transcription and apoptosis, protein kinase activity and DNA/RNA/protein binding activity.

**Table 2:** Main function groups of oncogene domains.

**Go annotation**	**Domain**
ATP binding	Pkinase, DEAD, Helicase_C, Pkinase_C, MutS_V, MutS_IV, MutS_III MutS_II MutS_I Furin-like, Ephrin_lbd

protein binding	zf-C3HC4, Death, LRR_1, PDZ, SAMP, EB1_binding, APC_basic, APC_15aa

DNA/RNA binding	Myb_DNA-binding, P53, bZIP_Maf, bZIP_2, RRM_1, zf-C2H2, zf-C4, bZIP_1, Ets, SAM_PNT

signal transduction	Cbl_N, wnt, C1_1, Death, RhoGAP, Ras

growth factor receptor activity	FGF, PDGF, PDGF_N, IGFBP

protein tyrosine kinase activity	Pkinase, Pkinase_Tyr, SH2, SH3, ig, I-sev, V-set

regulation of transcription	HLH, RHD, Wos2, Hormone_recep, zf-C4, bZIP_1, Ets, IRF, p53, Myc_N, WT1, E2F_TDP, Myc-LZ, bZIP_Maf, bZIP_2

regulation of apoptosis	BH, BH4

receptor factor activity/binding	Recep_L_domain, NCD3G, 7tm_1, 7tm_3, PSI

Wnt receptor signalling pathway	Wnt

zinc ion binding	zf-C2H2, zf-C3HC4, zf-C4, zf-RanBP

calcium ion binding	Cadherin, Cadherin_C

transcription factor activity	Hormone_recep, zf-C4, bZIP_1, Ets, RHD, IRF, P53, Myc_N, E2F_TDP, Myc-LZ

Further analyses suggest that domains with different functions might have come from different origins (Figure 1; Table 2). For example, domains related to immunoglobulin and tyrosine kinase (e.g., SH2, SH3, I-sev, and V-set) are found in archaea, bacteria or in both. These domains are known to be closely related to oncogenesis [16] (Note that another two important oncogenesis-related domains: Pkinase_Tyr and ig, originated in eukaryotes, but were horizontally transferred to prokaryotes [14]). Other domains such as rhodopsin domains (7tm_1, 7tm_3), cyclin dependent kinases (CDKs) domains (Cyclin_N, Cyclin_C) and the intracellular signalling domains (PH, CH) seem to have originated in eukaryotes. Several domains related to the development of the nervous system such as wnt, ephrin_lbd and Sema seem to have originated in metazoans. In addition, function domains required by vertebrates such as hormones involved in mitogenic and inflammatory activity (Myc_N, Myc_LZ, Maf_N, Cys_knot) seem to have originated in chordates.

#### Domains originated from viruses

Among the 103 identified oncogene domains, 38 are found to be present in viruses (Table 3). The three most frequently occurring domains in virus proteins are Helicase_C, Ank and DEAD. Ank has been reported in diverse groups of proteins such as enzymes, toxins and transcription factors. The existence of Ank in both prokaryotes and viruses may have resulted from horizontal gene transfers [17]. The Helicase domain family (including Helicase_C and DEAD) is reportedly related to hepatitis virus-associated hepatocellular carcinoma and involved in cell growth control [18]. In addition, some other families such as Zinc finger domains (zf-C3HC4, zf-C2H2, zf-C4), Immunoglobulin-related domains (ig, V-set, I-set) and protein-tyrosine kinase related domains (Pkinase_Tyr, SH2, SH3) also have remote homologs in viruses and all these three domain families are closely related to oncogenesis. Overall, 20 of the 38 virus-originated domains are known to be related to oncogenesis.

**Table 3:** 38 oncogene domains present in virus dataset (367,752 proteins).

**Num**	**Pfam accession id**	**Pfam entry id**	**Frequency of occurrence in virus proteome**	**Description of domain family**
1	PF00271	Helicase_C	1455	Helicase conserved C-terminal domain

2	PF00023	Ank	459	Ankyrin repeat

3	PF00270	DEAD	296	DEAD/DEAH box helicase

4	PF00097	zf-C3HC4	168	Zinc finger, C3HC4 type (RING finger)

5	PF00069	Pkinase	162	Protein kinase domain

6	PF00001	7tm_1	115	7 transmembrane receptor (rhodopsin family)

7	PF00047	ig	111	Immunoglobulin domain

8	PF07686	V-set	110	Immunoglobulin V-set domain

9	PF00048	IL8	87	Small cytokines (intecrine/chemokine), interleukin-8 like

10	PF00170	bZIP_1	50	bZIP transcription factor

11	PF01403	Sema	47	Sema domain

12	PF00167	FGF	33	Fibroblast growth factor

13	PF07714	Pkinase_Tyr	30	Protein tyrosine kinase

14	PF00096	zf-C2H2	22	Zinc finger, C2H2 type

15	PF00017	SH2	21	SH2 domain

16	PF00560	LRR_1	17	Leucine Rich Repeat

17	PF00018/PF07653	SH3	16	SH3 domain

18	PF00605	IRF	16	Interferon regulatory factor transcription factor

19	PF00041	fn3	15	Fibronectin type III domain

20	PF00341	PDGF	14	Platelet-derived growth factor

21	PF00452	Bcl-2	12	Apoptosis regulator proteins, Bcl-2 family

22	PF00134	Cyclin_N	11	Cyclin, N-terminal domain

23	PF01056	Myc_N	11	Myc amino-terminal region

24	PF00010	HLH	10	Helix-loop-helix DNA-binding domain

25	PF07679	I-set	8	Immunoglobulin I-set domain

26	PF02344	Myc-LZ	6	Myc leucine zipper domain

27	PF00076	RRM_1	5	RNA recognition motif

28	PF01437	PSI	5	Plexin repeat

29	PF02201	SWIB	5	SWIB/MDM2 domain

30	PF00104	Hormone_recep	3	Ligand-binding domain of nuclear hormone receptor

31	PF00105	zf-C4	3	Zinc finger, C4 type

32	PF07716	bZIP_2	2	Basic region leucine zipper

33	PF07988	Wos2	2	Mitotic protein Wos2

34	PF00071	Ras	1	Ras family

35	PF00595	PDZ	1	PDZ domain

36	PF00611	FCH	1	Fes/CIP4 homology domain

37	PF02757	YLP	1	YLP motif

38	PF04692	PDGF_N	1	Platelet-derived growth factor, N terminal

### B. Oncogene domain fusion

#### Domain fusion in cellular organisms

We have identified 50 whole domain fusion events in the 124 oncogenes. Among them, 21 contain two distinct domains (domain pairs) and the others contain at least three different domains. Their initial appearance in cellular organisms and their presence/absence in viruses are given [see Additional file 2].

#### Fused domains in viruses

Among the 50 fused domains, 7 events have been identified in viruses. These 7 fused domains can be divided into 4 categories according to their functions: pkinase-related domain fusion ({SH2, SH3, Pkinase_Tyr}, {SH2, FCH, Pkinase_Tyr}); platelet-derived growth factor domain fusion ({PDGF, PDGF_N}); helicases-related domain fusion ({DEAD, Helicase_C}) and DNA/ligand-binding domain fusion ({Hormone_recep, zf-C4}; {HLH, Myc_N, Myc-LZ}; {HLH, Myc_N}). Interestingly, ~90% of the virus proteins harbouring these fused domains come from the Potyviridae family and the remaining almost all come from the Orthoretrovirinae family. Potyviridae is one of the largest and most important families of plant viruses. Although the relationship between retroviruses and cancer has been widely established [19-21], the possible link between Potyviridae and oncogenesis is unknown.

### C. Proteome-wide patterns of origins of oncogenes

We have also examined the origins of all the oncogenes as a whole. Our goal is to find out at what stage in evolution all component domains of an oncogene are fused together for the first time, considered as the origin of the oncogene [see Additional file 3].

Among the 24 oncogenes whose initial domain fusions occurred in prokaryotes, 20 have the same domain fusions in viruses (Table 4). It seems that domains with prokaryotic origins tend to present in viruses.

**Table 4:** 24 oncogenes whose domain fusion events arose in prokaryotes.

**HUGO ID**	**Whole domain fusion events**	**Cellular origin of the whole domain fusion events**	**presence or absence in viruses of the whole domain fusion events**
MOS	{Pkinase}	A_B	P
SPINK1	{Kazal_1}	B	/
NRAS	{Ras}	A_B	P
HRAS	{Ras}	A_B	P
KRAS	{Ras}	A_B	P
GLI2	{zf-C2H2}	A_B	P
GLI3	{zf-C2H2}	A_B	P
MYBL2	{Myb_DNA-binding}	B	/
RALA	{Ras}	A_B	P
RALB	{Ras}	A_B	P
PIM1	{Pkinase}	A_B	P
CDK4	{Pkinase}	A_B	P
FOSL1	{bZIP_1}	A	P
TAL1	{HLH}	B	P
BCL3	{Ank}	A_B	P
DDX6	{DEAD, Helicase_C}	A_B	P
NKTR	{Pro_isomerase}	A_B	/
BMI1	{zf-C3HC4}	B	P
TGFBR2	{Pkinase}	A_B	P
MPL	{fn3}	A_B	P
MSH2	{MutS_V, MutS_I, MutS_II, MutS_IV, MutS_III}	A_B	/
RAB8A	{Ras}	A_B	P
MAX	{HLH}	B	P
EVI1	{zf-C2H2}	A_B	P

(A_B: archaea and bacteria; A: archaea only; B: bacteria_only; P: presence.)

We have divided the oncogenes into six categories according to their functions: signal transducers, no-receptor kinases, growth factors, growth factor receptors, transcription factors and others. Based on our examination of the oncogene origins, we have observed some general relationships between the origins and the functional categories of the oncogenes (Table 5).

**Table 5:** General classification of oncogene origins according to their functions.

**Oncogene classification according to their functions**	**Oncogenes**	**Origins**
signal transducers	*RALA, RALB, NRAS, KRAS, RAB8A, HRAS, CDK4, PIM1, MOS, NTRK1, TAL1, BCL3, MPL*	Prokaryotes
non-receptor kinases	*SRC, YES, FGR, HCK, LYN, FYN, LCK, ABL1, ABL2, FES, RAF1*	Metazoans
growth factors	*PDGFB*	Chordates
growth factor receptors	*EGFR*, *FGF3*, *FGF4*, *FGF6*, *FLT1*, *MET*, *PDGFB, ERBB2, ERBB3, ERBB4, WNT1, EPHA3, THPO, ROS1, KIT, RET*	Metazoans(9) Chordates(6)
transcription factors	*TP73, MAFG, MAF, TNF, JUN, MYB, MYBL1, MYCL1, MYCN, MYC, CXCL1, CXCL2, CXCL3, WT1, TIAM1, IRF4*	Metazoans(4) Chordates(12)
programmed cell death regulators	*BCL2, TP53BP2, CDH1, ABL1, BRAF, FGF4, DCC, LTA, LCK, TNF*	Metazoans

#### Signal transducers

In our dataset, most of the oncogenes acting as signal transducers originated from prokaryotes. We have observed that a large number of such genes contain the Ras and Pkinase domains, and are involved in signal transduction, protein binding and kinase activities. It is believed that most *ras *proteins exist in an inactive state in the resting cell where they bind GDP [22], and their oncogenesis is closely related to their interactions with other receptors.

#### No-receptor kinases

Non-receptor kinases oncogenes are mostly tyrosine kinases discovered through retroviral transduction and/or through DNA transfection that do not have a receptor-like transmembrane domain. These proteins are partly associated with the inner surface of the plasma membrane, and more related to cell differentiation than to proliferation. Another group of serine/threonine kinases such as *RAF1 *also belongs to this category. Our analysis shows that all the oncogenes of this group originated from metazoans.

#### Growth factors

Only one oncogene PDGFB (*sis*) is known to be a growth factor. This gene encodes one of the two polypeptide chains that together constitute PDGF, a platelet-derived growth factor domain. Our analysis shows that the PDGF domain generally originated in metazoans or chordates, and the corresponding oncogene first came into being in chordates.

#### Growth factor receptors

The *ERBB *oncogene family was originally isolated from chicken erthroleukemia, encoding an epidermal growth factor (EGF) receptor [1]. Several other oncogenes also encode proteins with a receptor-like domain, including *KIT *and *ROS *[1]. These oncogenes consist of an extracellular ligand-binding domain, a transmembrane domain and an intracellular domain. Our analysis results show that these genes generally originated in metazoans or chordates, representing important regulatory proteins involved in phosphorylation [23].

#### Transcription factors

Transcription factors are nuclear proteins that regulate the expression of their target genes. They typically belong to multi-gene families that share common DNA-binding domains such as zinc fingers. Our data shows that oncogenes acting as transcription factors mostly originated in chordates, and a few of them (25%) came from metazoans. It has been speculated that the pathologically activated form of these transcription factors no longer fulfils their physiological regulating functions but acts as a carcinogen [1,24].

Many oncogenes of this category have been identified in our dataset. One representative is *JUN*, which can bind tightly to other nuclear onco-proteins. In addition, a substantial portion of oncogenes in this category belongs to the *myc *gene family that is related to nuclear transcription and myeloblastosis. It has been reported that the *Myc *genes have been found in a wide variety of vertebrates, including mammals, birds, amphibians, and fish [25,26]. The myeloblastosis function in these oncogenes may have evolved in response to some specific needs by chordates.

#### Programmed cell death regulators

The first oncogene shown to regulate programmed cell death is *BCL2 *[27]. Several other oncogenes related to apoptosis have also been identified in our dataset. We found that these oncogenes often originated in metazoans. The mechanisms of apoptosis have not been fully elucidated, but previous studies indicate that the process of apoptosis is controlled by a diverse range of cell signals which may originate either extracellularly (extrinsic inducers) or intracellularly (intrinsic inducers) [1,27]. This type of complex cell signal network may be more active and required by metazoans.

### D. Frequent domains and domain pairs in oncogenes

#### Oncogene domain co-occurrence graph

**Figure 2:**
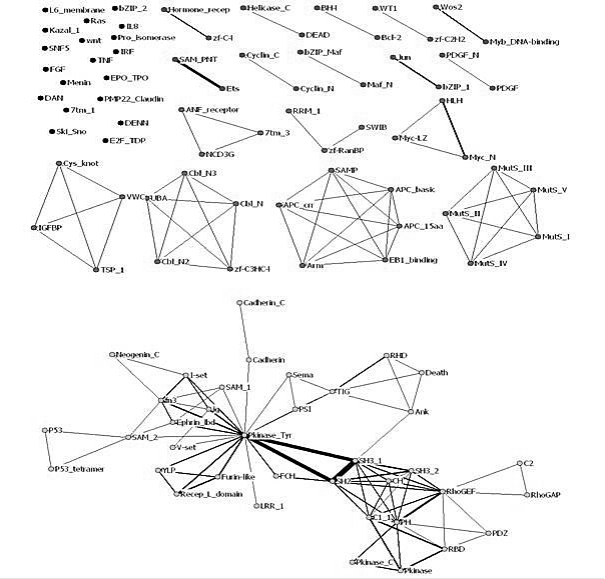
**Oncogene domain co-occurrence graph consisting of 105 domains**. Each node is labelled with a domain name. The weight of each edge represents the co-occurrence frequency across all the 124 oncogenes.

We have constructed a domain co-occurrence graph for 124 oncogenes, which consists of 105 domains (nodes) and 141 co-occurring domain pairs (edges), as shown in Figure 2. The graph has 8 connected components, each containing at least 3 nodes, with the largest component having 37 nodes and 82 edges. The graph has a sparse but highly clustered structure. The few highly connected nodes representing domains like Pkinase_Tyr, SH2, SH3 form hubs of the (co-occurrence) network.

**Figure 3:**
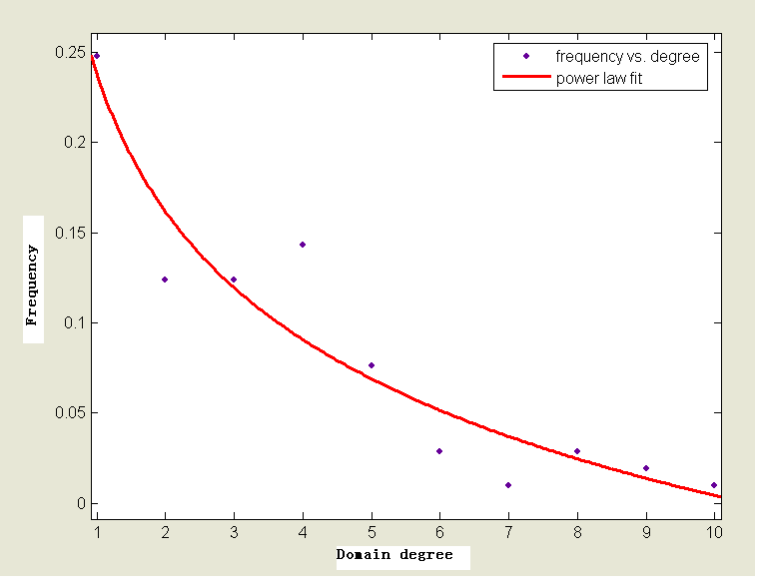
**Frequency distribution of node degrees in oncogene domain network**. The distribution follows a generalized power law:. Parameter values of the fit (solid curve) are a = 1.125; b = -0.887, and r = 0.101.

A large-scale analysis of co-occurrence networks of the protein domains collected from the ProDom, Pfam and Prosite domain databases was previously performed by S. Wuchty [28], which found that these networks exhibited small-world and scale-free properties. In our study, the same properties were observed for oncogene domain network (Figure 3). We conclude that the oncogene domain network has a sparse but highly clustered structure. Highly connected nodes emerge in the network which predominantly shapes the topology of the underlying network and a few domains are connected to many different domains forming a few hubs.

#### Frequent domains and domain pairs

We have identified a number of domains and domain pairs that are highly frequent in oncogenes, compared with those in the background human genome. We consider such domains as significant if they show high occurring frequencies and high numbers of co-occurring domains in the oncogenes but not in the background set. These domains are Pkinase_Tyr, SH2, SH3, RhoGEF and fn3, with functions related to signal transduction, enzymatic activity, and cell surface binding. Moreover, pkinase_Tyr has protein-tyrosine kinase activities, and SH2 and SH3 mediate protein interactions. They are known to play a key role in diverse biological processes such as growth, differentiation, metabolism and apoptosis in response to external and internal stimuli.

To find out which domain pairs occur more frequently in oncogenes than in the background genome, we have carried out enrichment analyses of the domain pairs. Table 6 gives the domain pairs with *p*-values more significant than 10^-6^. It should be noted that 10^-6 ^is a rather significant cut-off based on our experience in identifying frequent domain pairs from the background.

**Table 6:** Frequent domain pairs XY in the oncogene graph compared with the background genome.

**Number**	**Domain X**	**Domain Y**	***n***_*s*_	***m***_*s*_	**Enrichment ratio**	***p*-value**
1	SH2	SH3	63	16	51.25	6.84E-19
2	SH2	Pkinase_Tyr	41	11	54.15	7.83E-17
3	SH3	Pkinase_Tyr	32	9	56.76	3.42E-14
4	SAM_PNT	Ets	13	5	77.62	3.43E-09
5	Furin-like	Pkinase_Tyr	8	4	100.91	3.96E-08
6	Recep_L_domain	Pkinase_Tyr	8	4	100.91	3.96E-08
7	Furin-like	Recep_L_domain	10	4	80.73	1.18E-07
8	Jun	bZIP_1	3	3	201.81	1.19E-07
9	Pkinase	RBD	4	3	151.36	4.73E-07
10	C1_1	RBD	5	3	121.09	1.18E-06
11	Myc_N	HLH	5	3	121.09	1.18E-06
13	ig	Pkinase_Tyr	27	4	29.9	9.23E-06

(P-value cutoff is 10^-6^. n_s_: the number of proteins containing specific domain pair in the background genome. m_s_: the number of proteins containing specific domain pairs in the oncogene proteins. Background proteins set size: 25,025; oncogene proteins set size: 124)

We expect that domain fusions might have brought new functions to their host proteins. This type of functional transformation has been reported previously [25,29-31]. For instance, the SH3 and SH2 domains frequently appear together in various signalling proteins involved in recognition of phosphorylated tyrosine [30], where SH2 localizes tyrosine-phosphorylated sites and SH3 binds to target proteins [31]. Another example is that the bHLH motif and the "Myc boxes" co-exist in the *Myc *gene family. bHLH uses a common mechanism for DNA binding and dimerization while the *Myc *boxes, on the another hand, appear to be unique to the *Myc *family and are involved in transcription activation and neoplastic transformation [25]. While the individual functions of these two domains are generally understood, their synergistic effects in their bounded protein complex are not known [25].

Two significant triad domain fusions, {SH2, SH3, Pkinase_Tyr} and {Furin-like, Recep_L_domain, Pkinase_Tyr}, are found (Figure 2) and they form six fused domain pairs (shown in Table 6). Pkinase_Tyr are known to be related to protein tyrosine kinase activities and amino acid phosphorylation. The other two domains, Furin-like and Recep_L_domain, are involved in signal transduction by receptor tyrosine kinases [32]. It is also noteworthy that domains corresponding to the tyrosine kinase family are among the most frequent families in oncogenes. These domains may carry essential functions as standalone domains and may also extend their functionality to accomplish complex tasks in combination with other domains.

#### E. Phylogenetic profiling diversities of frequent domains and domain pairs

Diverse origins of frequent domains and domain pairs are found in cellular organisms through our phylogenetic profile analyses, which provide complementary information to our earlier analysis of domains and domain pairs. *Phylogenetic profiling *is a computational technique for functional analyses of domains and their fusions [33]. We have calculated the phylogenetic profiles of all oncogene domains and domain pairs to find their taxonomic distribution across 495 cellular genomes, grouped into 7 taxa: archaea, bacteria, protozoa, viridiplantae, fungi, metazoan-invertebrates, and metazoan-chordates. The phylogenetic profiles of frequent domains and domain pairs are listed in Table 7.

**Table 7:** Phylogenetic profiling analysis of frequent individual domains and domain pairs through 7 taxa from 495 genomes.

**Individual domain**	**Archaea**	**Bacteria**	**Protozoa**	**Viridiplantae**	**Fungi**	**Metazoan-invertebrates**	**Metazoan-chordates**
Phinase_Tyr	**-**	**+**	**+**	**+**	**+**	**+**	**+**
SH2	**-**	**-**	**+**	**+**	**+**	**+**	**+**
SH3	**+**	**-**	**+**	**+**	**+**	**+**	**+**
RhoGEF	**-**	**+**	**+**	**+**	**+**	**+**	**+**
fn3	**+**	**+**	**+**	**+**	**+**	**+**	**+**

**Domain pair**	**Archaea**	**Bacteria**	**Protozoa**	**Viridiplantae**	**Fungi**	**Metazoan-invertebrates**	**Metazoan-chordates**

SH2&SH3	**-**	**-**	**-**	**-**	**-**	**+**	**+**
SH2& Pkinase_Tyr	**-**	**-**	**+**	**-**	**-**	**+**	**+**
SH3& Pkinase_Tyr	**-**	**-**	**-**	**-**	**-**	**+**	**+**
SAM_PNT& Ets	**-**	**-**	**-**	**-**	**-**	**+**	**+**
Furin-like& Pkinase_Tyr	**-**	**-**	**-**	**-**	**-**	**+**	**+**
Recep_L_domain& Pkinase_Tyr	**-**	**-**	**-**	**-**	**-**	**+**	**+**
Furin-like& Recep_L_domain Pkinase_Tyr Recep_L_domain	**-**	**-**	**-**	**-**	**-**	**+**	**+**
Jun& bZIP_1	**-**	**-**	**-**	**-**	**-**	**+**	**+**
Pkinase& RBD	**-**	**-**	**-**	**-**	**-**	**+**	**+**
C1_1& RBD	**-**	**-**	**-**	**-**	**-**	**+**	**+**
Myc_N& HLH	**-**	**-**	**-**	**-**	**-**	**-**	**+**
ig& Pkinase_Tyr	**-**	**-**	**-**	**-**	**-**	**+**	**+**

(- for absence and + for presence)

Our data show that nearly all frequent individual domains originated in prokaryotes, and have a wide distribution across many genomes, while the frequent domain pairs almost all first emerged in metazoans (Table 7). Therefore, while individual domains may have early origins, most frequent domain pairs first came together in higher organisms. Although multi-domain proteins are more common in higher organisms, it is not clear if this observation about frequent oncogene domain pairs is generally true for any domain pairs from any groups of genes, which will be left for future study.

## Conclusion

We have analyzed the origins of component domains and domain fusions of oncogenes, and studied the unique characteristics of the oncogene domain pairs in comparison with those in the background human genome. Most of these domains and domain pairs are functionally related to protein tyrosine kinase activities, which are closely related to cancer pathophysiology. Our phylogenetic profile analysis provides additional evidence to support our observation that frequent domain pairs in oncogenes tend to originate in higher organisms. The knowledge gained from this computational study may provide useful insights about the complex processes of oncongenesis.

## Methods

### A. Data sources

124 proto-oncogenes of *Homo sapiens *were collected from CNIO OncoChip project website  and the Cancer Genome Anatomy Project database  [see Additional file 4], and their protein sequences were obtained from the Uniprot database [34] (only the primary protein form was used). The pre-calculated domain structures of these proteins were retrieved from the Pfam-A database (version 21.0) [8], using HMMER [8] and RPS-BLAST [8] (E-value cutoff 0.001; sequences were masked for coiled-coils and low complexity regions). Our list includes all the important proto-oncogenes previously reported in the literature [2,35-37]. All these proto-oncogenes were manually curated based on the published literature.

A proto-oncogene only becomes an oncogene when mutations or over-expressions take place [37]. Note that "oncogenes" are different from "cancer genes". Commonly we consider oncogenes as those involved in uncontrollable cell growth while cancer genes are generally referred to genes that are identified with somatic or germline mutations in cancer tissues. Futreal *et al*. recently conducted a census of human cancer genes on the basis of genetic evidence [16], whose cancer-gene list partly overlaps our oncogene list. Throughout the rest of the paper, we use oncogenes to refer proto-oncogenes for the terminology simplicity.

Two sets of genomes and their encoded proteins were used in our study, one including the whole set of proteins encoded in 495 sequenced genomes (with 34 archaea, 422 bacteria and 39 eukaryotes) from the Integr8 database (release 58) [38] and the other including 367,752 protein sequences with Pfam annotations from 6,774 sequenced virus genomes. The second data set was downloaded from the Uniprot database at the FTP site [39].

The complete set of proteins of *Homo sapiens *with Pfam domain annotation was downloaded from the Integr8 database, which contains 25,025 protein sequences without splicing isoforms. This dataset set served as the background for our statistical analyses.

It should be noted that currently there is no well-accepted benchmark dataset for oncogenes. Since our data were mainly selected from CNIO OncoChip project and Cancer Genome Anatomy Project database, a likely bias may exist when compared with other datasets. One future plan of our work is to investigate several other cancer gene datasets, including those identified by exon sequencing studies such as TCGA [40] dataset from the group at John Hopkins [41] and the cancer gene lists compiled by Futreal *et al*.[16], to derive a more comparative dataset of oncogenes.

### B. A computational pipeline for domain analyses of oncogenes

**Figure 4:**
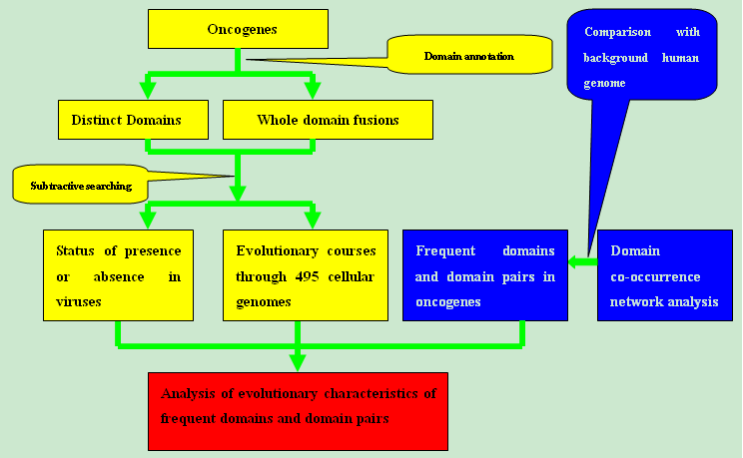
**A computational pipeline for prediction of origins of oncogene domains**. Different components of the pipeline are colour-coded with yellow for prediction of domain origins, blue for analysis of oncogene domain co-occurrence and red for analysis of evolutionary characteristics of frequent domains and domain pairs.

Our computational pipeline for identification of the origins of oncogene domains and domain fusion events consists of three main steps (Figure 4). The first step is to predict the origins of domains and domain fusion events in oncogenes, which is done through application of a *subtractive search *procedure [15], in conjunction with identification and analyses of horizontal gene transfers to avoid pitfalls, which could potentially lead to misclassification of domain origins in prokaryotes. The second step is to perform comparative analyses on domains between oncogenes and the background, namely the whole collection of human proteins. Domains and domain pairs with higher occurrence frequencies in oncogenes than in the background are identified, through an analysis of a domain co-occurrence graph. Detailed analyses on these domains and domain pairs are carried out in the third step of the pipeline, through a combination of a domain/domain pair enrichment analysis and a phylogenetic profile analysis (see following sections for details).

#### Subtractive search

**Figure 5:**
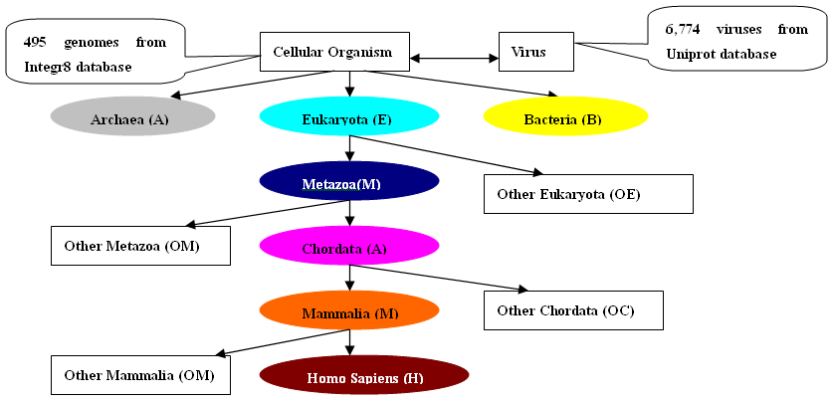
**A simplified taxonomy**. For cellular organisms, each ellipse represents a major taxonomic class. Each rectangle represents all organisms covered by its parent class but not covered under its sibling ellipse.

First we generate the domain list of all the 124 oncogenes, and search them against all the sequenced genomes, which are organized into a simplified taxonomy tree, including viruses, archaea, bacteria, eukaryotes, plus a few increasingly finer subclasses of eukaryotes leading to *Homo sapiens*, namely metazoans, chordates and mammalian (Figure 5). The questions we ask here are (a) for each domain, where did it occur for the first time going from a simplest class of organisms to the most complex one? (b) for each pair of co-occurring domains, where did the co-occurrence take place for the first time in the aforementioned taxonomy?

In Figure 5, the term *other_node *is used to denote the group of organisms excluding the next higher node in the taxonomy. For example, if node B is next to node A in the taxonomy, '*other_node*A' refers to all species from 'nodeA minus nodeB'. Thus, for node eukaryote, '*other_eukaryota*' refers to all species from eukaryotes minus metazoans. Briefly, the tracing procedure starts from the organisms in a bottom-level group, and goes up the taxonomy tree to higher organisms in each of the groups. It should be noted that when a remote homolog of a domain is found at one *other_node*, its node of origin will be its immediate lower major node. Then the hit domains will be subtracted from the set and the others will be searched against the higher level *other_node *until all *other_nodes *are searched along the whole taxonomy. For example, if a domain is found at the *other_chordata *node, then its node of origin will be the chordata node. When a domain does not have a hit when searched against all the *other_node *genomes, then its node of origin will be considered as *Homo sapiens *[15].

We have used three types of nodes of origin for bacteria and archaea depending on the presence of remote homologs, i.e. *archaea_only *(first hit only in archaea, but not in bacteria), *bacteria_only *(first hit only in bacteria but not in archaea) and *archaea_bacteria *(first hit in both archaea and bacteria).

A tool package *TaxDom *is developed to execute the procedure outlined above and to facilitate visualization of the search results. The program is written in Perl and Java.

#### Refinement of subtractive search results

Horizontal gene transfer (HGT) has played a substantial role in organismal and genome evolutions [42]. Gene transfer from prokaryote to eukaryotes, particularly in the context of organellar endosymbiosis, is a major evolutionary phenomenon [43]. However, horizontal transfer in the opposite direction, *i.e*., from eukaryotes to bacteria or archaea, has been reported only anecdotally [14]. Although this type of transfer may occur only rarely, we have performed manual curation on the tracing results generated by the *subtractive searching *procedure, to fix any false origination classification for proteins that have been reported in literature to have emerged in eukaryotes first and then were transferred to prokaryotes. We have corrected such false origination predictions by our above procedure, based on our extensive literature search results.

#### Domain co-occurrence graph and enrichment analysis

We define a *domain co-occurrence graph *[44] as follows. Each node represents a distinct domain in the oncogenes, and two nodes are linked by an edge if they co-occur in some proteins in one of the reference genomes. Each edge has a weight defined as the number of co-occurrences of the corresponding domains in the same protein. Note that this graph is not necessarily a connected graph.

The following defines the enrichment ratio [45] of domains between oncogenes and the background human genome, for identification of domain pairs with higher co-occurrence frequencies in oncogenes compared to the whole human genome. Let

*N *= the number of proteins in the background set,

*n*_s _= the number of proteins in the background that contain domain pair *s*,

*M *= the number of proteins in the oncogene set, and

*m*_s _= the number of oncogene proteins that contain domain pair *s*.

We use the following formula to calculate the enrichment ratio of proteins that contain a specific domain pair in oncogenes and its *p*-value, knowing that it follows a hypergeometric distribution [45]:

$$Enrichment\_ratio=\frac{m_{s}/M}{n_{s}/N}$$

$$p-\text{value}=\{\begin{array}{c} \sum_{k=m_{s}}^{n_{s}}\frac{\left(\begin{array}{c} M\\ k \end{array}\right)\left(\begin{array}{c} N-M\\ n_{s}-k \end{array}\right)}{\left(\begin{array}{c} N\\ n_{s} \end{array}\right)},enrichment\_ratio\geq 1\\ \sum_{k=0}^{m_{s}}\frac{\left(\begin{array}{c} M\\ k \end{array}\right)\left(\begin{array}{c} N-M\\ n_{s}-k \end{array}\right)}{\left(\begin{array}{c} N\\ n_{s} \end{array}\right)},enrichment\_ratio<1 \end{array}$$

## Availability and requirements

*TaxDom *is the computational tool that we developed for visualizing domain evolution and their fusion events presented in this study, and it is freely accessible at .

## Authors' contributions

QL carried out the design and implementation of the computational pipeline and drafted the manuscript. JH was responsible for the evolutionary analysis of the oncogenes. HL and PW participated in the preparation and analysis of oncogene data. YX and XY conceived the study and coordinated the involved data analyses as well as writing the manuscript. All authors read and approved the final manuscript.

## Supplementary Material

Additional file 1 **Supplementary S2.** 103 domains encoded from oncogene proteins. Click here for file

Additional file 2 **Supplementary S3. **50 whole domain fusions of oncogenes. Click here for file

Additional file 3 **Supplementary S4. **Proteome-wide patterns of origin nodes in oncogene proteins. Click here for file

Additional file 4 Supplementary S1. oncogene list. Click here for file
